# Identification and Verification of Candidate miRNA Biomarkers with Application to Infection with *Emiliania huxleyi* Virus

**DOI:** 10.3390/genes14091716

**Published:** 2023-08-28

**Authors:** Enquan Zhang, Shumiao Zhang, Guiling Li, Zhengxiao Zhang, Jingwen Liu

**Affiliations:** College of Ocean Food and Biological Engineering, Jimei University, No. 43, Jiyuan Road, Xiamen 361021, China; chriszhangen@163.com (E.Z.); zsm0128@163.com (S.Z.); guiling_li@hotmail.com (G.L.); zxzhang@jmu.edu.cn (Z.Z.)

**Keywords:** *Emiliania huxleyi*, *E. huxleyi* virus, miRNA, biomarker, dual-luciferase reporter assay

## Abstract

The interactions of *Emiliania huxleyi* and its specific lytic virus (EhV) have a profound influence on marine biogeochemical carbon–sulfur cycles and play a prominent role in global climate change. MicroRNAs (miRNAs) have emerged as promising candidates with extensive diagnostic potential due to their role in virus–host interactions. However, the application of miRNA signatures as diagnostic markers in marine viral infection has made limited progress. Based on our previous small-RNA sequencing data, one host miRNA biomarker that is upregulated in early infection and seven viral miRNA biomarkers that are upregulated in late infection were identified and verified using qRT-PCR and a receiver operating characteristic curve analysis in pure culture, mixed culture, and natural seawater culture. The host ehx-miR20-5p was able to significantly differentiate infection groups from the control in the middle (24 h post-infection, hpi) and late infection (48 hpi) phases, while seven virus-derived miRNA biomarkers could diagnose the early and late stages of EhV infection. Functional enrichment analysis showed that these miRNAs participated in numerous essential metabolic pathways, including gene transcription and translation, cell division-related pathways, protein-degradation-related processes, and lipid metabolism. Additionally, a dual-luciferase reporter assay confirmed the targeted relationship between a viral ehv-miR7-5p and the host dihydroceramide desaturase gene (*hDCD*). This finding suggests that the virus-derived miRNA has the ability to inhibit the host sphingolipid metabolism, which is a specific characteristic of EhV infection during the late stage. Our data revealed a cluster of potential miRNA biomarkers with significant regulatory functions that could be used to diagnose EhV infection, which has implications for assessing the infectious activity of EhV in a natural marine environment.

## 1. Introduction

*Emiliania huxleyi* (Haptophyta) is one of the most abundant coccolithophores, is widely distributed in the world’s oceans, and forms extensive blooms annually [1]. Importantly, *E. huxleyi* is also the key producer of calcite (CaCO_3_) [2,3] and dimethyl sulfide, a bioactive gas that significantly regulates climate by enhancing cloud formation [4,5]. Thus, the fate of *E. huxleyi* blooms may critically affect the carbon and sulfur cycles. It has been proven that the blooms formed by *E. huxleyi* are routinely terminated via programmed cell death (PCD) due to infection by a giant double-stranded DNA virus, *E. huxleyi* virus (EhV, ∼180 nm in diameter) [6,7]. Recent studies showed that infection with EhVs stimulated the release of transparent exopolymer particles in *E. huxleyi* [8], thereby facilitating the formation of sinking particles and enhancing the biological pump efficiency [9]. In addition, host cell lysis induces the release of dissolved organic matter into the water (the viral shunt) [10] and influences the efficiency of microbial carbon bumps [11]. Therefore, the interactions between *E. huxleyi* and the virus have a great impact on oceanic microscale ecosystems and biogeochemical cycles.

For diagnosing the level of active EhV infection in natural oceanic populations, it is necessary to identify functional biomarkers. Numerous studies demonstrated that EhV infection could lead to the reprogramming of glycerolipid and sphingolipid metabolism in host cells [12,13]; therefore, some lipid biomarkers were selected to diagnose the infection status, such as host sialic acid glycosphingolipids (sGSLs) and virus-specific GSLs (vGSLs) [14,15]. In addition, a set of chlorine–iodine-containing metabolites was detected in extracellular vesicles in virus-infected oceanic *E. huxleyi* blooms, which could be a distinct hallmark of EhV infection [16]. Most recently, our study revealed that both host and viral microRNAs (miRNAs) perform essential and diverse regulatory functions, especially in fatty acid and glycerolipid metabolism [17]. However, miRNA-related biomarkers have not been widely adopted as diagnostic tools for successful infection with EhVs.

miRNAs are single-stranded non-coding RNAs with a length of 19–24 nucleotides that complement the 3′ untranslated sequence of target mRNAs and regulate gene expression at the post-transcriptional level [18,19]. The interaction between miRNAs and mRNAs can lead to the degradation or inhibition of mRNA translation [20]. In addition to the traditional diagnostic marker molecules, such as mRNAs, proteins, and lipids, miRNAs have emerged as promising candidates with significant diagnostic potential due to their essential roles in post-transcriptional gene regulation, simplified structures, specific expression, and easily quantified and highly stable characteristics [21]. Recently, an increasing number of studies have indicated the application of miRNAs as novel diagnostic markers for viral infectious diseases [22]. Kawano et al. (2013) first reported an altered miRNA expression pattern in patients suffering from chronic active Epstein–Barr virus (CAEBV) infection and that the signature miRNAs could serve as potential biomarkers for assessing disease severity or prognosis [23]. Since then, the ability of microRNAs to serve as biomarkers of hepatitis C virus (HCV) [24], HIV [25], and influenza A virus infection [26], as well as numerous other infectious conditions, has been evaluated. Given the essential regulatory role of miRNAs in virus-infected *E. huxleyi* that we previously identified, we hypothesize that certain miRNAs may also serve as novel diagnostic signatures for EhV infection. 

In the present study, significantly differentially expressed miRNAs (including both host and viral miRNAs) were screened from infected *E. huxleyi* cells during the entire infection process (0, 6, 12, 24, 48, and 60 h post-infection, hpi) and verified by qPCR. Then, these miRNAs were further validated by qPCR in pure culture, mixed culture (including seven strains of coccolithophores), and natural seawater culture. Finally, a dual-luciferase reporter assay was performed to confirm the potential target relationship between a viral miRNA and its target gene. Our data presented a cluster of potential miRNA biomarkers with important regulatory functions that could be used to diagnose EhV infection.

## 2. Materials and Methods

### 2.1. E. huxleyi Strain and Virus Preparation

*E. huxleyi* BOF92 was isolated from the North Sea at 48° N, 12° W, and the *E. huxleyi* virus (EhV) 99B1 was isolated from the Norwegian Fjords at 60° 24′ N, 5° 19′ E [27]. *E. huxleyi* was cultured in a f/2-Si medium at 16 ± 0.5 °C under a light intensity of approximately 100 µmol quanta m^−2^s^−1^ with a 14:10 h light–dark cycle. Exponentially growing cells were infected with EhV99B1. Once the host culture was cleared (5–6 days later), the lysate was passed through a GF/F with 0.45 μm and 0.22 μm filters. The filtrate was concentrated 50 times with a tangential ultrafiltration system (Prep/Scale TFF-1, PTQK50, Millipore, MA, USA). Virus enumerations were counted using flow cytometry. The concentrates were diluted from 1:10 to 1:1000 in a TE buffer (10 mM Tris, 1 mM EDTA, pH 8.0) and stained for 10 min at 80 °C with a commercial SYBR Green-1 solution (Molecular probes, Eugene, OR, USA) at a final concentration of 10^−4^ of the commercial solution. The samples were analyzed at a flow rate of 50–100 events per second, and the green fluorescence signal was recorded. The concentration of EhVs was up to ~1 × 10^7^ virions per mL, and the concentrates were stored at 4 °C.

### 2.2. miRNA Biomarker Candidate Identification

Based on our previous sequencing data [17], potential host miRNA biomarkers were screened using the following criteria: (1) compared with the control groups, the fold change of miRNA expression was greater than 3; (2) miRNA sequencing abundance was consistent with qRT-PCR results; (3) expression was detected in all samples; and (4) in biological replicates, the coefficient of variation of miRNA expression was less than 1. For viral miRNAs since viral miRNAs were only expressed in the infected samples, potential viral miRNA biomarkers could be identified if they were expressed in all biological replicates.

### 2.3. Culture System Setup and Sampling

To assess the potential application of virus-derived miRNAs as biomarkers for quantifying active infection in complex natural marine environments, multiple infection cultures were performed to verify the specific expression of candidate miRNA markers in the EhV-*E. huxleyi* systems. Exponentially growing cultures were infected with viral concentrates in a volume ratio of 1:50 (EhV:Eh). Different cultures were set up as follows: Pure cultures: The *E. huxleyi* BOF92 strain was cultured and infected with EhV99B1 (different batch with miRNA-Seq). Mixed cultures: Seven strains of coccolithophores, including *E. huxleyi* BOF92, *E. huxleyi* CCMP2090, *E. huxleyi* CCMP1516, *E. huxleyi* BO, *E. huxleyi* PMLB92/11, *E. huxleyi* CS369, and *Pleurochrysis carterae* LAMB143, were infected with EhV99B1. Natural seawater cultures: The *E. huxleyi* BOF92 strain was cultured and infected with EhV99B1, and the f/2-Si medium was prepared with unpasteurized natural seawaters (containing other microbes).

In the case of host miRNAs, control and infected samples were collected at 6 hpi (early stage), 24 hpi (middle stage), and 48 hpi (late stage) in the pure culture system. An additional 72 hpi (post-infection) sampling point was added to the mixed culture and natural seawater culture system. As for viral miRNAs, since there was none of the expression abundance of viral miRNAs in our sequencing data at 0–24 hpi [17], infected samples were collected only at 24 hpi, 48 hpi, and 72 hpi (or 60 hpi). Each sample was collected with 100 mL cultures and was set up in six biological replicates.

### 2.4. Quantitative Real-Time PCR and Receiver Operating Characteristic Curve (ROC) Analysis

The cDNA products of host miRNA ehx-miR20-5p were synthesized using a miRNA 1st Strand cDNA Synthesis Kit (by stem-loop) (Vazyme, Nanjing, China), while the cDNA products of viral miRNAs were synthesized by a miRcute Plus miRNA First-Strand cDNA Kit (TIANGEN, Beijing, China). These cDNA products were subsequently utilized as the templates for qPCR. All qPCR reactions were carried out using the Universal SYBR Green Supermix (Vazyme, Nanjing, China) in 96-well plates in a Roche LightCycler 480II/96 Real-time PCR System (Roche, Switzerland) according to the manufacturer’s recommendations. The relative abundance of miRNAs was normalized to U6 (small nuclear RNA) and calculated using the 2^−ΔΔCT^ method [28]. All experiments were performed in triplicate. The primers used for qPCR are listed in Table 1.

### 2.5. Prediction and Function Enrichment Analysis of miRNA Targets

To predict the genes targeted by differentially expressed miRNAs, miRanda-3.3a [29] algorithms were used to predict the target sites of miRNAs and then searched for matches of miRNA seed region (nucleotides 2–8 from the 5′end of the miRNA) with a binding energy ≤ −20 kcal/mol and score ≥ 140. KEGG pathway enrichment analysis was performed for the predicted targets of the miRNAs using the DAVID online tool [30]. The *p*-value was calculated using a hypergeometric test and corrected by Q-value. A Q-value < 0.05 was considered a significant enrichment.

### 2.6. Dual-Luciferase Reporter Assay

Dihydroceramide desaturase (*DCD*) is one of the enzymes participating in ceramide synthesis via the de novo pathway and is a potential molecular target for regulating PCD. To investigate the potential targeting relationship between the viral miRNA ehv-miR7-5p and its predicted potential target gene, host *DCD* (*hDCD*), a dual-luciferase reporter assay was conducted to check ehv-miR7-5p binding to its downstream target. The partial 3′UTR of the *hDCD* gene (~400 nt) containing the predicted target site of ehv-miR7-5p was synthesized and cloned into the psiCHECK-2 vector (Promega, Madison, WI, USA). As a control, a sequence containing the mutated ehv-miR7-5p target sites was also cloned into the same vector. Ehv-miR7-5p mimics, mimics NC (negative control), *hDCD* 3′UTR, and mutated segments were synthesized by Sangon Bioteach (Shanghai, China). All recombinant plasmids were confirmed by sequencing. miRNA transfection experiments were performed in HepG2 cells. Briefly, the cells were seeded in 24-well plates with a 1 mL medium without antibiotics until 60–80% confluence and then co-transfected with 20 pmol ehv-miR7-5p mimics/mimics NC and 0.6 µg wild-type/mutated constructs using 3 µL Lipofectamine 2000 (Invitrogen, Carlsbad, CA, USA) per reaction. The luciferase activities in the cell lysates were measured forty-eight hours after transfection using the Dual-Luciferase Reporter Assay System (Promega, Madison, WI, USA), following the manufacturer’s instructions. The firefly luciferase activity was normalized to that of the Renilla luciferase.

### 2.7. qPCR of DCD Genes

Pure cultures: The *E. huxleyi* BOF92 strain was cultured and infected with EhV99B1. At 0, 6, 12, 24, 48, and 60 hpi, 100 mL of culture were collected by centrifugation at 2500× *g* for 5 min at 4 °C. The algal cell pellets were stored at −80 °C for subsequent total RNA extraction. Each sample was set up in three biological replicates. The TRIzol Reagent kit (Invitrogen, Carlsbad, CA, USA) was used for the total RNA extraction following the manufacturer’s instructions. First-strand cDNA was synthesized with 2 μg of total RNA using the GoScript Reversed Transcription reagent kit with gDNA Eraser (Promega, Madison, WI, USA) in a 20 μL reaction according to the manufacturer’s protocol. The cDNA samples were stored at −80 °C for subsequent analysis.

qPCR was performed to determine the changes in gene expression of *hDCD* and viral *DCD* (*vDCD*) genes. The primers used for qPCR were designed by Primer Premier 5 and listed as follows: *hDCD*: F-5′ CGGAGTGGCGGTCAAAGTA-3′, R-5′CGGCGACTTGAAGAAGAGGT-3′; *vDCD*: F-5′AAAGAACAACCGATAGACACCG-3′; R-5′GGGATTGAATGACGATTAGGAGT-3′. The gene templates of *hDCD* and *vDCD* used for primer design were obtained from our previous de novo transcriptome [17]. qPCR was performed using the Universal SYBR Green Supermix (Vazyme, Nanjing, China) in 96-well plates on a Roche LightCycler 480II/96 Real-time PCR System (Roche, Switzerland) according to the manufacturer’s instructions. The cyclin-dependent protein kinase A gene (*CDKA*) was used as an internal reference to calibrate the expression level of *DCD* genes in the EhV host system [31]. The comparative threshold (2^−ΔΔCt^) method was used to calibrate the relative gene abundance. All experiments were performed in triplicate.

### 2.8. Statistical Analysis

SPSS 25.0 software (SPSS Inc., Chicago, IL, USA) was used to perform statistical analysis. The triplicate qPCR data are shown as the mean ± SD, and a significant difference was evaluated by Student’s *t*-test. A difference at *p* < 0.05 was considered statistically significant.

## 3. Results

### 3.1. The Potential miRNA Biomarker Candidates

**Figure 1:**
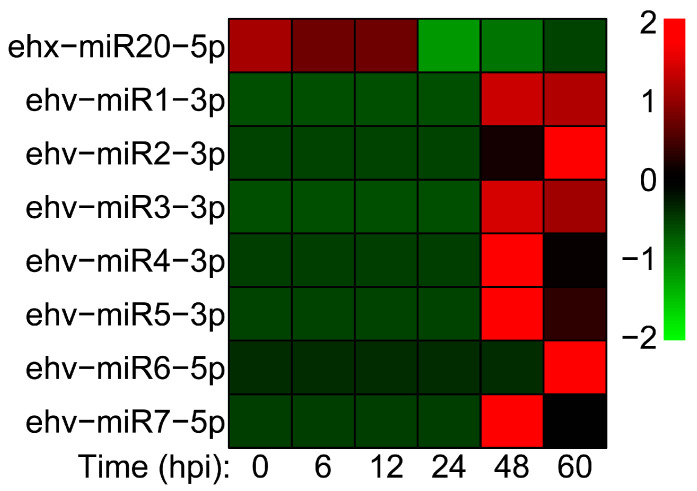
The heatmap of seven miRNAs during virus infection (based on our previous miRNA-Seq data).

Based on our previous miRNA-Seq data [17] and the screening criteria for miRNA biomarkers described in the method section, one host miRNA and seven viral miRNAs were identified as potential biomarker candidates. The expression trends of these miRNAs are exhibited in Figure 1. Ehx-miR20-5p (derived from *E. huxleyi*) showed a relatively high expression during the early stage of viral infection (0–12 hpi), suggesting that it could be used as a biomarker for early EhV infection. On the contrary, all six viral miRNAs showed high expression levels at 48 and 60 hpi, indicating that these virus-derived miRNAs could be severed as biomarkers for late infection. The expression levels of these seven miRNAs were verified by qRT-PCR in our previous study. The results obtained from miRNA-Seq were found to be in agreement with the qRT-PCR results, as previously reported [17]. 

### 3.2. Verification of miRNA Biomarker Candidates in Different Culture Systems by qRT-PCR

**Figure 2:**
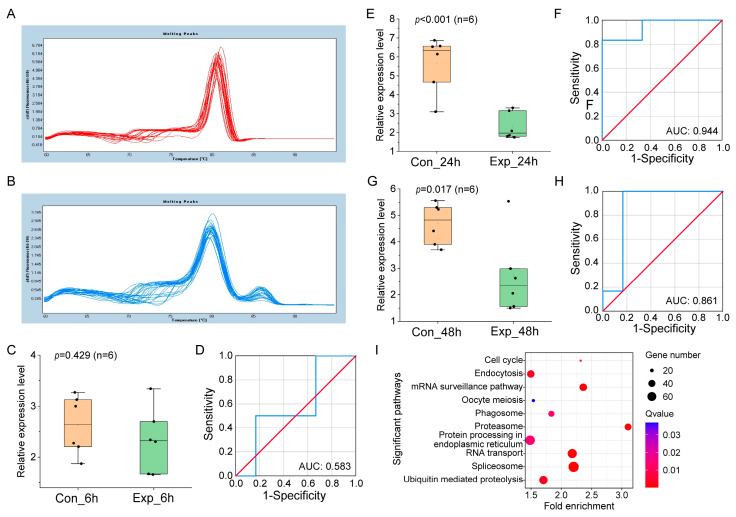
The validation of miRNA biomarkers in pure culture. (**A**,**B**) Melting curves of reference gene U6 and ehx-miR20-5p, respectively. (**C**,**E**,**G**) show the relative expression level of ehx-miR20-5p in control and infected groups at 6, 24, and 48 hpi. (**D**,**F**,**H**) show the ROC analysis of the results in (**C**,**E**,**G**), respectively. The blue line is the ROC curve. The red line is the random classifier line. (**I**) KEGG enrichment analysis of targets of ehx-miR20-5p.

Both host and viral miRNA biomarker candidates were verified in pure culture, mixed culture, and natural seawater culture using qRT-PCR. In the pure culture, the melting curves of the reference genes U6 and ehx-miR20-5p both displayed single peaks (Figure 2A,B), indicating the high amplification specificity of qPCR. Ehx-miR20-5p was not differentially expressed in infection groups at 6 hpi compared to control groups (*p* = 0.429, Figure 2C), and the area under the curve (AUC) value in ROC analysis was only 0.583 (Figure 2D). In contrast, ehx-miR20-5p were significantly downregulated at 24 hpi (*p* < 0.001, Figure 2E) with a high AUC value of 0.944 (Figure 2F). The expression of ehx-miR20-5p at 48 hpi was similar to that at 24 hpi (*p* = 0.017, AUC = 0.861, Figure 2G,H). These results indicate that ehx-miR20-5p can significantly distinguish control and infection groups at the middle (24 hpi) and late infection (48 hpi). The target gene enrichment results of ehx-miR20-5p are shown in Figure 2I. Significantly enriched pathways (Q < 0.05) included Endocytosis and Phagosome, indicating that viruses enter algal cells by Endocytosis at the late stage of infection. In addition, there were pathways involved in gene transcription and translation, such as spliceosome, the mRNA surveillance pathway, RNA transport, and protein processing in the endoplasmic reticulum, indicating that gene transcription and protein synthesis were active during and after viral infection, which is conducive to viral replication and assembly. Furthermore, cell division-related pathways such as cell cycle and oocyte meiosis were also significantly enriched, indicating that some algal cells might form haploids through meiosis to escape from viral infection [32]. Finally, there were several protein degradation-related processes, such as ubiquitin-mediated proteolysis and proteasome, which might be associated with programmed cell death [33,34].

**Figure 3:**
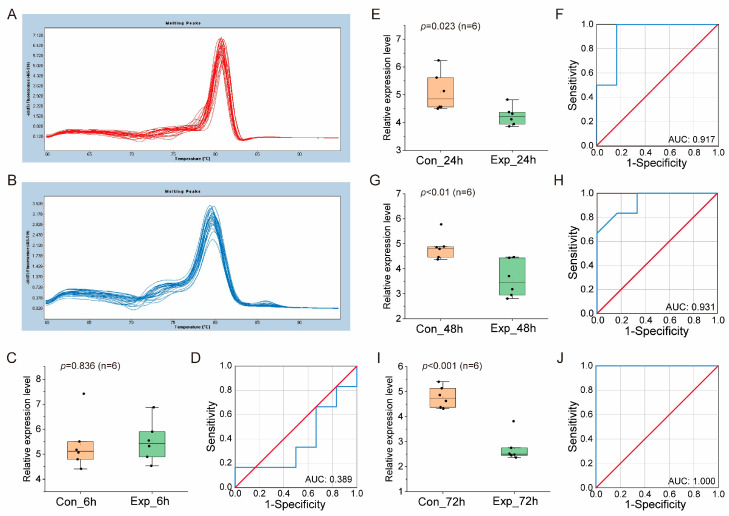
The validation of miRNA biomarkers in mixed culture. (**A**,**B**) Melting curves of reference gene U6 and ehx-miR20-5p, respectively. (**C**,**E**,**G**,**I**) show the relative expression level of ehx-miR20-5p in control and infected groups at 6, 24, and 48 hpi. (**D**,**F**,**H**,**J**) show the ROC analysis of the results in (**C**,**E**,**G**,**I**), respectively. The blue line is the ROC curve. The red line is the random classifier line.

**Figure 4:**
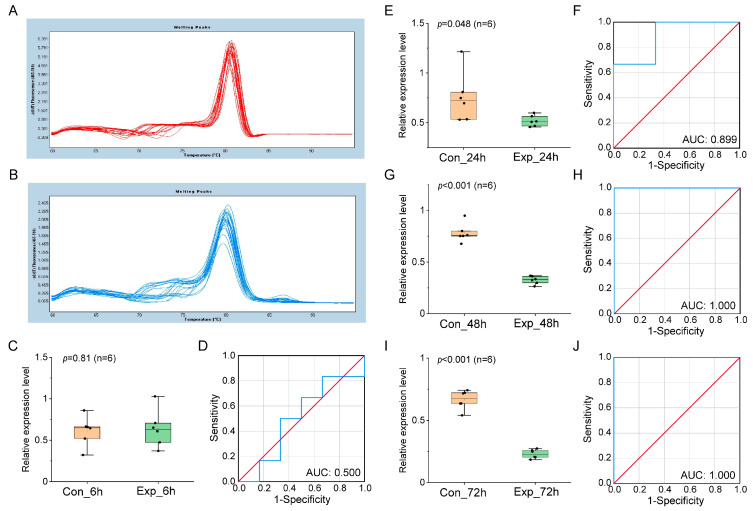
The validation of miRNA biomarker in natural seawater culture. (**A**,**B**) Melting curves of reference gene U6 and ehx-miR20-5p, respectively. (**C**,**E**,**G**,**I**) show the relative expression level of ehx-miR20-5p in control and infected groups at 6, 24, and 48 hpi. (**D**,**F**,**H**,**J**) show the ROC analysis of the results in (**C**,**E**,**G**,**I**), respectively. The blue line is the ROC curve. The red line is the random classifier line.

The expression of ehx-miR20-5p was validated as well in mixed culture (Figure 3) and natural seawater culture (Figure 4), which was largely compatible with the results obtained in pure culture, further confirming the reliability of ehx-miR20-5p as a downregulated miRNA biomarker at the middle and late infection stage.

**Figure 5:**
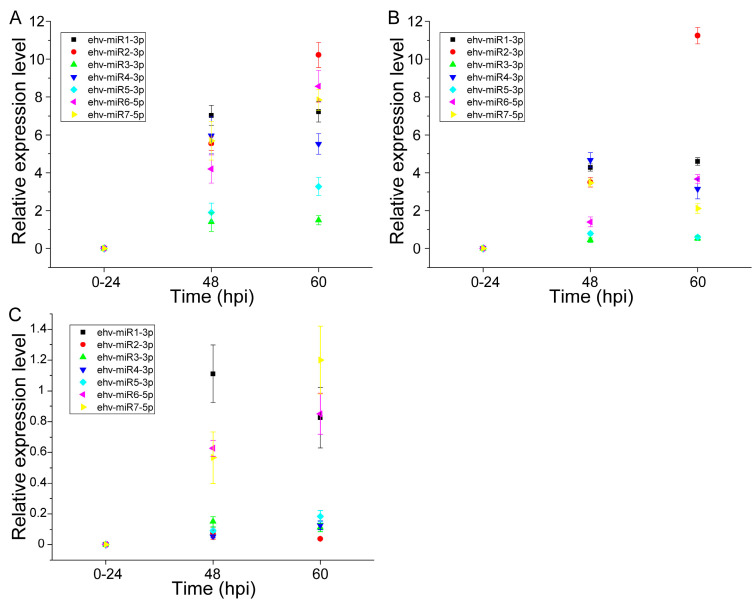
The validation of virus-specific miRNA biomarkers. (**A**–**C**) show the relative expression level of seven viral miRNAs in pure culture, mixed culture, and natural seawater culture, respectively.

As for virus-derived miRNAs, compared to the expression levels at 24 hpi, the qRT-PCR analysis revealed that all seven of these miRNAs showed significant upregulation at 48 and 60 (or 72) hpi in pure (Figure 5A), mixed (Figure 5B), and natural seawater (Figure 5C) culture. The upregulation of viral miRNAs during late infection may be involved in the regulation of sphingolipid metabolism, steroid biosynthesis, terpenoid backbone biosynthesis, autophagy, ABC transporters, ubiquitin-mediated proteolysis, etc. [17].

### 3.3. ehv-miR7-5p Could Target the hDCD Gene

**Figure 6:**
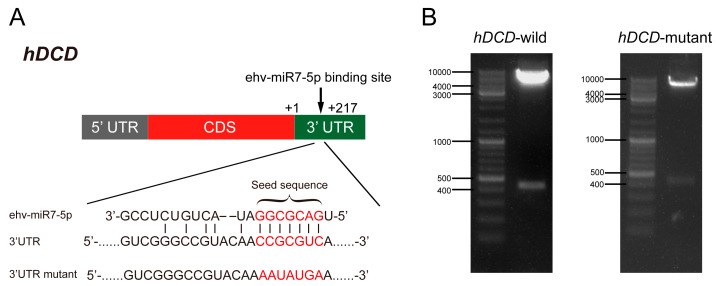
Interactions between miRNAs and their target genes. (**A**) Putative binding sites of ehv-miR7-5p on the 3′UTR region of *hDCD* and mutations were displayed in red. (**B**) The double enzyme digestion experiment results of recombinant vector psiCHECK2 (*hDCD* wild-type and mutant-type vectors).

**Figure 7:**
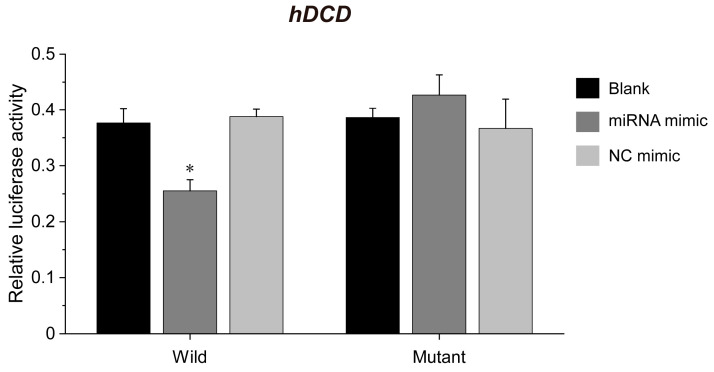
The relative luciferase activity in *hDCD*-3′UTR luciferase reporter assay. Blank groups were used as controls. The results were analyzed by Student’s *t*-test. * *p* < 0.05.

**Figure 8:**
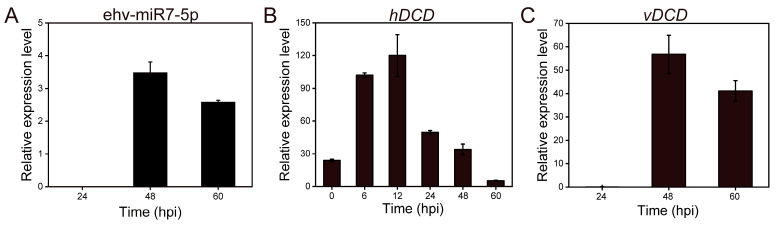
qRT-PCR results of ehv-miR7-5p and *DCD* genes. (**A**) ehv-miR7-5p. (**B**) *hDCD* gene. (**C**) *vDCD* gene.

Based on the bioinformatic prediction of differentially expressed miRNA target genes in virus-infected *E. huxleyi* cells, we found that viral ehv-miR7-5p targeted the *hDCD* gene with perfect pairing in the 3′-UTR [17]. The specific binding sites between ehv-miR7-5p and *hDCD* were further verified by the dual-luciferase reporter assay (Figure 6). As shown in Figure 6A, the seed sequence of ehv-miR7-5p is completely paired with the 3′-UTR of *hDCD*. The 3′-UTR sequences of *hDCD* were further inserted into the psiCHECK2 plasmid to construct a wild-type recombinant vector (WT vector). The 3′-UTR seed sequences were mutated to construct a mutant-type recombinant vector (MT vector) (Figure 6A). The wild-type and mutant psiCHECK2 recombinant plasmid constructs were then validated using restriction enzyme digestion (Figure 6B) and DNA sequence analysis. The results of the dual-luciferase reporter assay indicated a significant decrease of 34.3% in the psiCHECK2 *hDCD* WT vector and miRNA co-transfected group, compared to the *hDCD* MT vector and miRNA control mimic-transfected sample (*p* < 0.05). However, there was no significant difference observed in the results of transfection with the mutant vector (Figure 7). Moreover, the expression levels of *hDCD and vDCD* genes were further verified by qPCR (Figure 8), displaying a negative correlation between ehv-miR7-5p and *hDCD* (Figure 8A,B). It means that ehv-miR7-5p might inhibit the expression of *hDCD* during late infection. In contrast, vDCD upregulated significantly during late infection (Figure 8C), consistent with the metabolic switch toward viral sphingolipid biosynthesis. These results suggest that the *hDCD* gene might be a target of ehv-miR7-5p and that ehv-miR7-5p may be involved in the regulation of EhV-induced PCD by targeting *hDCD*.

## 4. Discussion

The interactions between *E. huxleyi* and EhV are characterized by a lipid-based coevolutionary “arms race” [1]. This process involves host lipid metabolism remodeling, viral sphingolipid production [12], host oxidative stress [35], programmed cell death [7], and so on. The entire viral infection process lasts for about 72 to 96 h [36]. Therefore, functional biomarkers that can distinguish viral infection status are needed to diagnose active infection levels in natural marine populations and quantitatively assess their ecosystem and biogeochemical impacts. At present, the screening of biomarkers for the characterization of EhV infection mainly focuses on the screening and identification of lipid metabolites. Significantly upregulated lipid biomarkers during viral infection include betaine-like lipids BLL (22:6/22:6) [14], viral sphingolipid vGSLs [37], host TG (57:8), TG (53:3), TG (64:17), FA (20:4) [15], etc. Significantly downregulated lipid biomarkers include sialic acid GSLs (sGSLs) [14], Cer (38:1; 2), Cer 40:2; 2_isomer1 [15], etc. In addition, host or virus-specific mRNA can also act as biomarkers, such as the host cytochrome oxidase C subunit 1 (*COI*) gene [9], the thylakoid membrane protein-coding gene *psbA* [38], and the viral capsid protein-coding gene *MCP* [38]. In this study, based on miRNA sequencing results, one host (ehx-miR20-5p) and seven viral miRNA biomarkers were screened, which could distinguish the early and late infection stages, respectively. As a kind of small non-coding RNA, the detection of miRNAs is more accurate, simple, and specific, which has become an emerging method to diagnose mammalian virus infection [21]. The miRNA biomarkers identified in this study further expand the biomarker repertoire of the *E. huxleyi*-EhV system and provide a scientific reference for quantifying the impact of marine viruses on microbial food webs, especially for assessing the contribution of *E. huxleyi* blooms to marine carbon export [39].

Functional enrichment analysis of ehx-miR20-5p target genes showed that the ehx-miR20-5p could target cell cycle and meiosis (Figure 2I). Previous studies have shown that oxidative stress induced by coccolithovirus infection can activate PCD to prevent virus replication [40] or induce a life cycle shift from the susceptible diploid to the resistant flagellated haploid life phase [32]. A recent study found that virus-infected *E. huxleyi* has the capability to secrete miRNAs through extracellular vesicles, which could also target the host cell cycle and meiosis processes [41]. Therefore, it is hypothesized that endogenous miRNAs induced by viral infection may participate in the regulation of the host’s transition from diploid to haploid by directly regulating intracellular-related target genes or by functioning as signal molecules in the form of extracellular vesicles.

The dual-luciferase reporter assay results indicated that ehv-miR7-5p could target *hDCD* through binding to its 3′-UTR and significantly inhibited *hDCD* gene expression (Figure 8), suggesting that virus-derived ehv-miR7-5p could inhibit host sphingolipid metabolism in late infection. *DCD* is an enzyme participating in ceramide synthesis via a de novo pathway. It can catalyze the insertion of a 4,5-trans-double bond to the sphingolipid backbone of dihydroceramide, resulting in the conversion of dihydroceramide into ceramide [42]. Ceramides are one of the most studied sphingolipids due to their complicated role in cell differentiation, death, and stress response [43]. Other previous work has highlighted that host sphingolipid biosynthesis was inhibited during EhV infection on both transcriptional and metabolic levels [12]. Recently, we further confirmed that viral infection led to a shift toward virus-specific sphingolipids, which is consistent with the downregulation of genes involved in the host de novo sphingolipid biosynthesis, such as serine palmitoyltransferase (*SPT*), ceramide synthetase (*CERS*), and *DCD* genes [44]. These results demonstrate that the sphingolipid biosynthesis process in virus-infected *E. huxleyi* is not only transcriptionally but also epigenetically regulated. Interestingly, we revealed the possible regulatory role of miRNA in glycerides and fatty acids metabolism through the PI3K-Akt-TOR signaling pathway in the EhV host system [17]. Thus, the EhV-mediated miRNA regulation might indicate that marine viruses have evolved to regulate the expression of their host genes by miRNA silencing to meet their lipid metabolic requirements.

Taken together, the eight miRNA biomarkers identified in this study as biomarkers of viral infection were able to characterize the entire process of viral infection (including early, middle, and late infection) and had potential applications for assessing the infectious activity of EhVs in a natural marine environment.

## Figures and Tables

**Table 1:** Oligonucleotide primers used for miRNA qPCR.

miRNA ID	Primer Sequences
U6-F	CTCGCTTCGGCAGCACA
U6-R	AACGCTTCACGAATTTGCGT
ehx-miR20-5p	CGGCGGTAGTCGGCGGTAAA
ehv-miR1-3p	GGAATTGGTCGTCACGTTGTTGT
ehv-miR2-3p	GGTGAGAGTGCATCGGATTGTGAA
ehv-miR3-3p	GGATTTGGGCTGGCCCAAAA
ehv-miR4-3p	GGATCTAGGAAAGATTGAGGCCAAA
ehv-miR5-3p	GGCGAAGACACTGTGAATCAAGT
ehv-miR6-5p	CGGGCGGAAAATATGATTCGTTA
ehv-miR7-5p	GCCTGACGCGGATACTGTCTC

## Data Availability

All other data generated during this study are included in this published article.
